# Hydroxyapatite granules used in the obliteration of mastoid cavities in rats

**DOI:** 10.1590/S1808-86942011000300008

**Published:** 2015-10-19

**Authors:** Rogério Hamerschmidt, Rafael Francisco dos Santos, João Cândido Araújo, Henrique Jorge Stahlke, Miguel Angelo Agulham, Ana Tereza Ramos Moreira, Marcos Mocellin

**Affiliations:** 1 MSC, professor at the UFPR; 2 Specialist, Physician at the Otorhinolaryngology Department - University Hospital - Federal University of Paraná (UFPR); 3 PhD, Professor - Department of Surgery - University Hospital - Federal University of Paraná; 4 PhD, Professor - Department of Surgery - University Hospital - Federal University of Paraná; 5 PhD, Professor - Department of Surgery - University Hospital - Federal University of Paraná; 6 PhD, Head of the Department of Ophthalmo-Otorhinolaryngology - University Hospital - Federal University of Paraná; 7 PhD, Full Professor of Otorhinolaryngology - University Hospital - Federal University of Paraná; Hospital de Clínicas - Universidade Federal do Paraná - Departamento de Oftalmo-Otorrinolaringologia e Departamento de Cirurgia

**Keywords:** hydroxyapatites, mastoid, bone regeneration

## Abstract

Prospective Experimental study in which we created a bony defect in the mastoids of rats and filled it up with hydroxyapatite to evaluate bone regeneration, to solve the problems of open cavities after mastoidectomies that frequently present with otorrhea, infection, granulation tissue and hearing loss.

**Objective:**

The aim was to evaluate bone regeneration in defects created in the mastoids of rats, using hydroxyapatite, to see how much of the cavity we could reduce.

**Material and methods:**

Twelve rats Wistar-Furth were used. A 0.5 × 0.5 cm bone defect was created in both temporal bones of the rats, and filled with 15 micrograms of hydroxyapatite. The left side was used as control. The animals were slaughtered 40 days afterwards and histology analyses were carried out.

**Results:**

In the hydroxyapatite group, the new bone growth involved an area of 68.53% of the total; and in the control group it was only of 15.97%.

**Discussion and Conclusion:**

It was observed a very good hydroxyapatite integration to the temporal bone in this experimental model. The microscopic results were superior with the use of hydroxyapatite when compared to the control group. It is a safe method and easy to apply to solve the problems of open cavities with chronic discharge and difficult to clean.

## INTRODUCTION

Numerous biomaterials with the capacity to regenerate bone are being tested, materials such as calcium hydroxyapatite and tricalcium phosphate^1^. All these synthetic porous substitutes share advantages when compared to auto and allografts, including their easy sterilization and storage, let alone their unlimited availability. Among their disadvantages, we list: fragile handling, different degrees of resorption, poor performance in diaphysis defects and potential adverse effects on bone remodelling^2^. Regarding autologous bone grafts, disadvantages include limited quantity, inadequate resorption and high morbidity on donor site, thus the constant investment in bone graft technology^3^.

Synthetic calcium hydroxyapatite (SCH), [CaIO(PO_4_) (OH)], is an inorganic material used in bone gaps and a component of the mineral phase of calcified tissues. Its synthetic equivalent has biocompatibility and osteoconduction, which place it among the most important bone substitutes available today. It can be used in bone defects without load or in gaps in which load, torsional or shearing stress are neutralized by stiff implants, such as plates and screws.

Bone regeneration brought about by SCH has been studied in different animal and human models. Its first use in animal models was in canine proximal tibia defects, where the researchers noticed a fast graft take after the implant, no adverse effects and high bone regeneration^4^.

Bone conduction happens when a porous structure is implanted on the bone or near it. Fibrovascular tissues, capillaries and bone-forming cells migrate to the porous structure and start forming new bone. The success of such mineral grafts is based on their resorption by osteoclasts, which contribute to forming new bone. The non-decalcified bone graft is gradually and slowly absorbed by osteoclasts, which open gaps in the calcified bone, enabling vascular growth and the inflow of inflammatory cells, which explains how this tissue can maintain its mechanical force^5^.

Moreover, the collagen present in these bone substitutes make up the substrate for mineralization and promotes osteoinduction. The properties to adsorb molecules are also clear, promoting an excellent support for the longstanding action of anti-cancer drugs in tumor processes, such as osteosarcomas^6^.

There are contraindications for the use of this type of material in intraarticular fractures, in which one uses autologous grafts because of their adhesiveness capacity. Nonetheless, it has been successfully used in osteochondral defects in femoral condyles of rabbits^7^.

Based on all these principles, we started to try to use it in the temporal bone, because since it has respiratory mucosa areas, it behaves very differently from other bones. Our main focus was turned to the mastoid portion of the temporal bone, because it is the one most involved in chronic otitis, especially cholesteatomas.

Cholesteatomas can be defined as the presence of skin in the middle ear cavity and mastoid - places where usually there is only respiratory mucosa, thus not sites which are ready to bear keratinized epithelium, from the ventilation standpoint^8^.

It has an external matrix made up of keratinized stratified squamous epithelium on a fibro-connective tissue, which sheds itself in lamellae within the space it outlines, filling out and promoting local distension and bone erosion.

Cholesteatomas are traditionally classified into congenital or acquired, and they can be primary or secondary.

Clinically, the congenital cholesteatoma grows insidiously, manifesting itself as progressive conductive hearing loss. Meanwhile, acquired cholesteatomas are associated with migration to the middle ear, attic retraction in the primary or tympanic perforation in the secondary, and the pathogenesis of the acquired cholesteatoma is directly associated with middle ear diseases. The treatment for such disorder is primarily surgical, and its basic goals are: to eradicate the disease, to dry the middle ear and hearing conservation. Surgery is tympanomastoidectomy, which creates a cavity that is often times large, without proper ventilation through the external ear canal, and causes some problems such as hearing loss, otorrhea and the very discomfort of not being able to wet one's ear. This cavity is epithelized in the months that follow the surgery, and there is more skin forming on top of the bone throughout the cavity. The larger the cavity, the larger must be the meatoplasty, because if the skin surface is enlarged, the ventilation on this skin must also be greater, so as to avoid forming granulation tissue, secretion and consequently, otorrhea. Therefore, two solutions have been tried, one would be to increase ventilation, with increasingly larger and less esthetic meatuses, or surface reduction, in other words, the same size of the cavity, so as to have less epithelial tissue formed coating the cavity^9^.

## OBJECTIVES

The goals of the present study were:

1. to assess the degree of bone regeneration in a defect created on the mastoid of rats using synthetic calcium hydroxyapatite, establishing which percentage of the mastoid cavity can be reduced, by means of histological analysis under light microscopy;

2. to analyze whether or not there is new bone formation induction caused by synthetic calcium hydroxyapatite.

## MATERIALS AND METHODS

In carrying out this study we complied with all the guidelines from the Brazilian College of Experimentation with Animals (COBEA). The research project was approved by the Ethics in Research with Animals from the Health Sciences Sector of the institution under registration # AN.016.007.08.11, on December 10, 2008.

### Animals used

We used 12 Wistar-Furth adult rats, weighing between 180-220 grams. They were kept in a proper environment, with natural lighting and temperature, and fed with *ad libitum* water and food.

### Animal anesthesia

**Figure 1:**
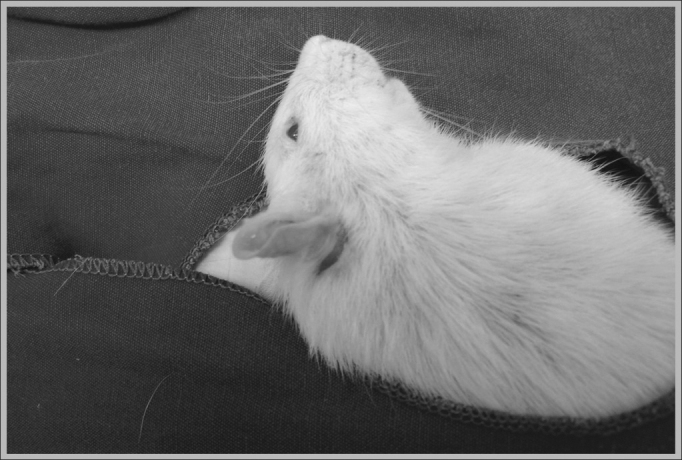
Behind-the-ear incision in a rat used in the experiment.

**Figure 2:**
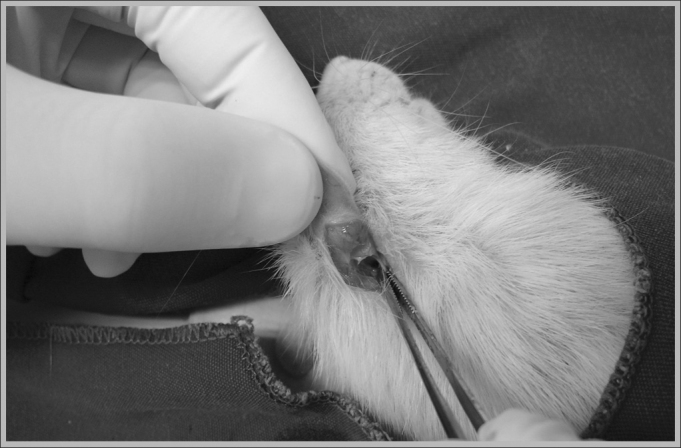
Exposure of the external ear canal and mastoid.

The animals were anesthetized through intramuscular injection, for which we used Ketamine 40 mg/kg, Diazepam 2 mg/kg and Butorphanol 2 mg/kg, and the doses were repeated during the procedure, according to the need, then the retroauricular access was used (Figure 1) for exposure of the external ear canal and the mastoid (Figure 2).

### Bone defect creation

**Figure 3:**
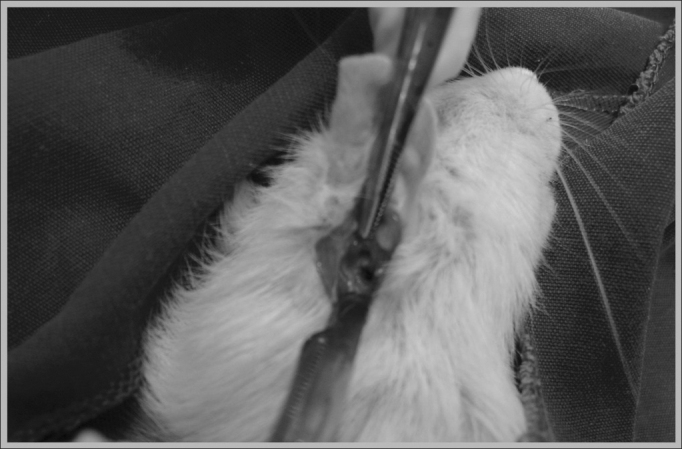
Bone defect creation on the mastoid which will be filled out by hydroxyapatite.

A 0.5cm × 0.5cm bone defect on the mastoid was created on all the animals with a round # 7.5 Sorensen® low rotation burr (Figure 3). The filling out of the bone defect was standardized on the right side with 15 micrograms of Genius Gen-phos® SCH in granules, which enables ventilation and gas exchange, registered at Anvisa #10345500004, and on the left it was not filled out, and it served as a control.

Antibiotic-prophylaxis was used during surgery with a dose of cephazolin 0.01 ml/kg. The animals remained alone in cages, with controlled lighting and *ad libitum diet. Post-operative analgesia was done with* 2mg/kg subcutaneous ketoprofen BID during 7 days.

### Slaughtering

The animals were slaughtered on the 40th day of post-op, through the administration of a lethal dose of barbiturates, according to what was advocated on Ordinance 876 from February 15, 2008 by the Federal Board of Veterinary Medicine (CFMV), because at the time of the slaughtering, the animals had a higher weight, around 500g, and therefore, neck breaking was no longer the method of choice for slaughtering animals with this body weight.

### Area morphometry and histological analysis

**Figure 4:**
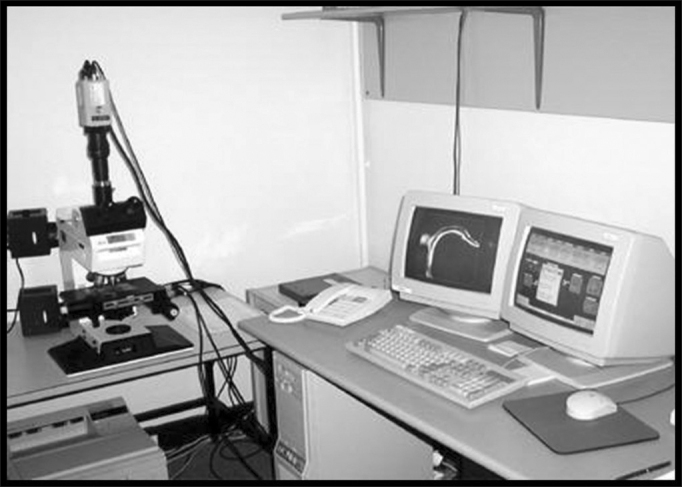
Equipment used for area morphometric studies.

**Figure 5:**
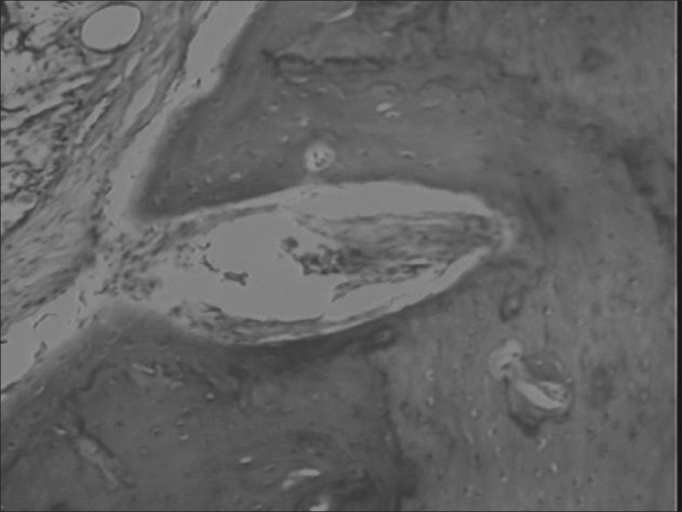
Histological analysis of the hydroxyapatite group.

We then carried out a microscopic analysis, and the slides were dyed by hematoxylin-eosin (HE) and evaluated by an objective analysis by means of the area morphometry (Figure 4), we considered new bone formation and total area porosity, in other words, the area which was not obliterated by SCH and by new bone formation (Figure 5). The area morphometry was studied by means of the pro-Image Plus v. 4.5 for Windows® software, coupled to a Sony video camera and an Olympus BX 50 microscope, calibrated with a 10x magnification lens, using the area morphology application through the color difference between bone tissue and connective tissue. Through this difference in color, the program called Area Morphology calculates how much of the total area was filled out by hydroxyapatite, and the values were statistically processed in comparison to the control group.

### Statistical test utilized

In the histological analysis (percentage of porosity of new bone formed) we employed the binomial test, with the values given by the software. We fixed at 5% (*p*< 0.05) the null hypothesis rejection level (level of significance), in other words, the likelihood of the result being found by chance.

## RESULTS

Concerning the clinical analysis, all the animals remained still and did not have complications in their postoperative period. No animal had infection nor die during the follow up until they were slaughtered on the 40^th^ day.

There was also no case of Dura-mater perforation during surgery, and this would take the animal off the study, because the cavity would have its anatomical boundaries altered, and the animal would probably die. In relation to microscopic analysis, we assessed the presence of a local inflammatory process, there was monocyte infiltrates in 31.44% of the temporal bone grafted with hydroxyapatite and in 35.31% of the control group. The neutrophil infiltration was present in both groups in minor proportions, which characterizes a chronic local inflammatory process. This inflammatory process happened to both groups; therefore, we stress the fact that it was not higher in the hydroxyapatite group, showing that the graft does not increase the neutrophil infiltrate, which already happens simply because of the normal healing process.

There was no foreign body granuloma formation because of the biocompatibility already proven with calcium hydroxyapatite, and also no case of extrusion.

We noticed the presence of bone fragments in 5.26% of the temporal bones grafted with hydroxyapatite, and in 1.13% of the control group, corresponding to the new bone formation induced by hydroxyapatite. These new bone formation areas may, with time, cause an even greater reduction of cavity size, because the osteoblastic activity remains for undetermined time.

As far as the area morphometry is concerned, we found in the control group (without graft) new bone formation in 15.97% and the porosity remained, in other words, the area which was not occluded by the graft, in 84.02% in average; therefore, corresponding to the greater part of the area of the bone defect created.

In the hydroxyapatite group, the new bone formation makes up for an area corresponding to 68.53% of the total (p: 0.0022), with porosity persistence around 31.47% (Table 1), making the mastoid cavity size reduction with hydroxyapatite statistically significant in comparison to the control group, when only the mastoid was opened.

**Table 1:** New bone formation analysis in the area morphometry in the control and hydroxyapatite groups.

BONE NEW FORMATION POROSITY GROUP
CONTROL (N=12) 15.97% 84.02%
HYDROXYAPATITE (N=12) 68.53% 31.47%
SIGNIFICANCE P=0.0022

## DISCUSSION

The synthetic calcium hydroxyapatite is approved for use with humans by the *Food and Drug Administration* (FDA), and it has been used in daily medical practice in numerous situations^10^. It has been showing excellent biocompatibility - producing new bone formation, little fibrosis with moderate inflammatory reaction and it does not produce a foreign body reaction^11^.

In 1991, Takahashi described the use of SCH to fill up the mastoid^12^. The results described using hydroxyapatite associated with a muscle flap in open cavities is excellent^13^. The author reports that hydroxyapatite is safe and useful to avoid the problems of mastoid cavities and equally to prevent the recurrence of cholesteatomas. Often times, very large open cavities require an unaesthetic extensive meatoplasty and they frequently form granulation tissue and skin shedding which must be cleaned frequently so as to avoid reinfection and also to reduce the likelihood of cholesteatoma recurrence. Muscle flaps alone have a very large resorption and necrosis rate, which makes the cavity reduction not very efficient. The use of hydroxyapatite alone or in combination with the flaps can minimize this problem, because the bone incorporation of hydroxyapatite is very good. Another important detail is associated with the very porosity of hydroxyapatite, a primordial item, being the mastoid a porous bone which requires ventilation. Therefore, the bone covered by hydroxyapatite is not totally without ventilation, which reduces the risk of cholesteatoma formation underneath the material used for filling or synthetic material, in this case, hydroxyapatite.

Portmann & Portmann^14^ have already described the V/S ratio, in other words, the volume/surface ratio, which shows that the larger the epithelized surface of the mastoid cavity, the greater must be the air volume penetrating the cavity, thus the need for a meatoplasty to enable cavity ventilation. On the other hand, there is the possibility of keeping this ratio, not only increasing the amount of air entering, but also reducing the epithelized surface area, reason for starting to use hydroxyapatite.

Bone graft take has 5 stages: 1) inflammatory, promoting a host cell response; 2) tissue revascularization; 3) osteoconduction, in which the graft has the function of being a framework for the growth of vessels and bone formation; 4) osteoinduction, the host's mesenchymal cells are induced to turn into osteoblasts by proteins found in the graft and 5) bone remodeling with the characteristics of continuous bone formation and resorption.

Cholesteatoma recurrence in closed cavities may happen in five situations:

1 through the attic defect,
2 through erosion on the bone wall,
3 by pars tensa invagination,
4 by invagination between an attic defect and the normal pars tensa,
5 by lamellae of cholesteatomas left in the mastoid cavity, especially in the facial nerve recesses and in the tympanic sinus. It is for this reason that patients submitted to canal-wall-up tympanomastoidectomies, in other words, preserving the posterior wall of the external ear canal, must be controlled by computerized tomography, and usually done after one year of surgery.

Black et al.^11^ reported the use of a semicircular block of hydroxyapatite in the ear attic region in order to reduce cholesteatoma recurrence, creating reinforcement on the tympanic membrane pars flacida, which reduces the possibility of retraction and consequent cholesteatoma formation. This surgical step is already routinely done with the use of cartilage from the ear pinna or the tragus; nonetheless, this cartilage is resorbed with time and this enables retraction pocket recurrence.

Kveton et al. reported on the use of hydroxyapatite in skull base reconstructions, including surgical accesses through the translabyrinthine path, via middle suboccipital cranial fossa. Fifteen of their patients were followed up during two years and they did not have complications^15^. The goal in these cases is to fill up the cavity created by the surgical access, which is usually done with abdominal fat, which also has a high rate of resorption^16^, ^17^, ^18^.

Matic et al. reported some contraindications concerning the use of hydroxyapatite.^19^ The main one would be doubt about whether or not the cholesteatoma has been completely eradicated because of the occlusion of the cavity by any autologous graft, or it would create a barrier against ventilation and foster cholesteatoma recurrence, especially if some microscopic cholesteatoma matrix would remain after surgery. In this case, since hydroxyapatite is porous, the risk is much smaller than that with a muscle flap, which completely blocks mucosa ventilation in this cavity, and, therefore, increases the likelihood of disease recurrence.

Other authors, such as Hussain et al. confirmed this hypothesis and already consider that hydroxyapatite is porous and enables the ventilation of the mucosa underneath the graft, even if some epidermal lamellae remain, the cholesteatoma would not recur, stressing the gas exchange properties of this graft, thus favoring its use.^20^

In the cholesteatomas which extend to beyond the borders of the attic, reaching the antrum or the anterior attic region or when associated to important changes to the residual mucosa, such as granulation tissue, cholesterol granulomas and hyperplasia, it is preferable, according to Cruz et al. to perform a canal-wall-down mastoidectomy with sound conduction system reconstruction and surgical cavity reduction, when needed^14^. Cavity reduction, with occlusion of the mastoid tip and sinodural angle with hydroxyapatite reduces the need for post-operative maintenance, with a better cosmetic and technical result in the meatoplasty. To remove or not to remove the tip of the mastoid depends on the final size of this cavity, but it is very likely that through the use of hydroxyapatite alone, there would be enough reduction, making it unnecessary to surgically ablate the mastoid tip.

However, the filling up procedure must be done with hydroxyapatite granules and not the one in the form of cement, according to KVETON, because when used, the latter fosters post-operative infection. The hydroxyapatite cement does not have the technical characteristic of enabling gas exchange such as the granule form.

In the present study we could notice that the caustic hydroxyapatite is a biocompatible allograft, which promotes the proper temporal bone fitting. We did not see foreign body granuloma formation, congestion or abundant neutrophil infiltration. We only noticed moderate monocytes infiltration, characterized by a local moderate chronic inflammatory reaction, which also happened to the control group, being a normal reaction associated with local fibrosis and healing, and not because of the use of an allograft - here: hydroxyapatite. We did not have any case of graft extrusion, although there are reports of extrusion when used to reconstruct the ossicular chain, which is used in the form of cement, therefore with less biocompatibility and less bone assimilation. Longer studies can be done in order to better assess this possible complication.

The microscopic analysis tried to assess the morphology of the region where the defect was created, observing the abundant bone neogenesis in 68.53% of the defects created on the bone, therefore showing that a reduction in the mastoid cavity size can be reached, according to literature data, with the hydroxyapatite graft, statistically significant when compared to the control group, opening up a major field for ear surgery, especially that of chronic otitis. Thus there would be a reduction in the epithelized surface of the cavity, which reduces granulation tissue and otorrhea arising from insufficient ventilation; therefore, providing a more adequate surgical result and greater patient satisfaction. Moreover, there is less sound scattering; thus, bringing about less hearing sequelae caused by surgery. Obviously, there are numerous factors associated with the degree of hearing loss after surgery, especially ossicular chain damage caused by the cholesteatoma-induced bone erosion. Moreover, there is less sound scattering; therefore, greater vibration stimulus in the new eardrum, with lower likelihood of auditory sequelae because of surgery. Obviously, there are factors associated with the degree of hearing loss after surgery, especially the damage to the ossicular chain caused by the cholesteatoma-induced bone erosion, because of the release of osteolytic substances in the secretion formed when there is an infection in the epidermal lamellae. Therefore, the filling up of the mastoid cavity with its consequent volumetric reduction cannot, in itself, guarantee hearing improvement, there must be concurrently an ossicular chain reconstruction, with the ossicles themselves, as it is usually done, or through titanium prosthesis available in the market for the different degrees of erosion of this ossicular chain. Associating ossicular reconstruction with the volumetric reduction of the cavity, the auditory result tends to be satisfactory, and normal hearing may even be restored in some cases. With the binomial - statistical test, employed, which is a non-parametric test, used when there is only one variable, comparing two samples, and with the significance level set at 0.05, the result found had a p-value of 0.0022; therefore, the likelihood that the observed fact happens when the null hypothesis is true, it is much lower than the level of significance, making the results presented in terms of mastoid cavity size reduction with hydroxyapatite reliable and confirmed. The statistical test, through inductive logic, confirmed the research's hypothesis.

Future studies must be carried out for better assessment of these possibilities in human beings, since in the literature the number of scientific papers on the topic is still small, and the statistically significant results in human beings will serve to prove the success of the hydroxyapatite use with this goal in mind, since alone the muscle flaps did not prove efficient to solve this type of postoperative complication, and so far there is no other synthetic material with the same properties as hydroxyapatite which can play such role in chronic otitis surgery.

Moreover, the fact that the osteoblastic activity happened for an undetermined period of time may happen beyond bone neogenesis induction, also neo-angiogenesis, which would hold possible the increase in osteoblastic activity with time, making the cavity work with gas exchanges and no anatomical sequelae caused by the surgery. This can be confirmed with longer prospective studies, with the goal of analyzing whether the cavity's volumetric reduction continues to increase with time, and also through the microscopic analysis, looking for new vessel formation.

Regardless of all these factors in the long run, considering new bone formation and especially neo-angiogenesis, only the fact that in the short period of time of this study, 40 days, there had already been an important volumetric reduction of the mastoid cavity with excellent allograft take, opens up a major possibility for evolution in this type of surgery in regards of immediate and late postoperative morbidity, and also a significant reduction on the sequelae caused by surgery, chronic otorrhea and hearing loss, significantly increasing patient satisfaction, not only because of disease cure, but also because of the functional results. It is obvious that its use in human beings must be carefully analyzed, since this study was carried out in healthy animals, with normal and well-ventilated mucosa, and also because there are just a handful of studies confirming its use in human beings, but it still opens a major possibility for the future.

## CONCLUSIONS

1 We noticed a satisfactory integration of the porous hydroxyapatite to the temporal bone in this experimental model, with important reduction in the size of the mastoid cavity created.
2 There is bone neogenesis induction with consequent percentage reduction of this cavity. Future studies may confirm its potential use in human beings with diseased mucosa and little ventilation, opening up a major possibility for solving the problem of open cavities with chronic otorrhea and hearing loss.
